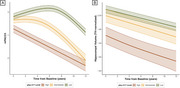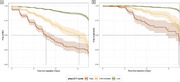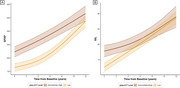# Long‐term cognitive and biomarker trajectories of cognitively unimpaired individuals with different levels of plasma ptau‐217

**DOI:** 10.1002/alz70856_105414

**Published:** 2026-01-07

**Authors:** Jesús Silva‐Rodríguez, Linda Zhang, Luca Kleineidam, Cristina Sánchez, Elizabeth Valeriano‐Lorenzo, Francisco J. López‐González, Sonia Wagner, Teodoro del Ser, Michel J. Grothe, Pascual Sánchez‐Juan

**Affiliations:** ^1^ CIEN Foundation, Reina Sofia Alzheimer Center, ISCIII, Madrid, Madrid, Spain; ^2^ University of Bonn Medical Center, Dept. of Neurodegenerative Disease and Geriatric Psychiatry/Psychiatry, Bonn, Germany; ^3^ CIEN Foundation, Reina Sofía Alzheimer Centre, ISCIII, Madrid, Madrid, Spain; ^4^ CIEN Foundation/Queen Sofia Foundation Alzheimer Center, Madrid, Spain; ^5^ Neurology Department, Hospital Universitario Marqués de Valdecilla – IDIVAL – University of Cantabria ‐ CIBERNED, Santander, Cantabria, Spain

## Abstract

**Background:**

Plasma ptau‐217 has demonstrated excellent performance in detecting AD pathology. However, its value for predicting long‐term cognitive decline among cognitively unimpaired (CU) individuals remains unclear.

**Method:**

We analyzed data from 1044 CU older individuals (75±4yrs, 64% female) enrolled in the Vallecas Project at the CIEN Foundation (Madrid, Spain). Participants underwent annual assessments (2011‐2024), including blood sampling, clinical and neuropsychological evaluations, and MRI scanning (average follow‐up: 7.2±3.0yrs). Plasma ptau‐217 levels were measured using the LUMIPULSE platform (Fujirebio^®^). Subjects were categorized as having either “*Low*” (<0.167pg/mL), “*Intermediate*” (0.167‐0.334 pg/mL) or “*High*” (>0.334 pg/mL) baseline ptau‐217 based on a pre‐established two cut‐off strategy. Generalized additive models were used to assess trajectories of (a) cognitive performance, assessed using a modified preclinical Alzheimer's cognitive composite score (mPACC5); (b) hippocampal volume, measured on serial MRI using SPM12/CAT12; and (c) blood biomarkers of astrocytic activation (GFAP) and neurodegeneration (NfL) (Simoa^®^ platform; *n* = 506). Additionally, Cox survival analysis was performed to assess the risk of progressing to MCI or dementia. All models were adjusted for sex, baseline age, APOE4, and years of education.

**Result:**

17.6% of the participants were classified as having “Intermediate” ptau‐217 levels, while 10.4% were classified as “High”. Subjects in the “High” group had lower baseline mPACC5 scores compared to the “Low” and “Intermediate” groups (d>0.5, *p* <0.001). Both the “High” and “Intermediate” groups showed accelerated cognitive decline compared to the “Low” group (Δslope > ‐0.3 points/year, *p* <0.001; Fig‐1A), and ptau‐217 was a strong predictor of conversion to MCI (“High” vs “Low”: HR=7.7; “Intermediate” vs “Low”: HR=3.3; *p* <0.001; Fig‐2A) and dementia (“High” vs “Low”: HR=14.3; “Intermediate” vs “Low”: HR=4.7; *p* <0.001; Fig‐2B). The “High” group showed smaller baseline hippocampal volumes (Δ∼5%, *p* <0.004) and accelerated atrophy rates compared to the other groups (Δslope = ‐0.3%/year, *p* < 0.01; Fig‐1B). Finally, the “Intermediate” and “High” groups showed elevated baseline GFAP (+39%, *p* <0.001) and NfL (+44%, *p* <0.02) levels, but progression did not differ between groups (*p* >0.33, Fig‐3).

**Conclusion:**

Our findings support the role of plasma ptau‐217 as a valuable biomarker for the early identification of CU individuals at risk for AD. Even individuals with intermediate values face an increased long‐term risk.